# Diphtheria in Lao PDR: Insufficient Coverage or Ineffective Vaccine?

**DOI:** 10.1371/journal.pone.0121749

**Published:** 2015-04-24

**Authors:** Naphavanh Nanthavong, Antony P. Black, Phonethipsavanh Nouanthong, Chanthasone Souvannaso, Keooudomphone Vilivong, Claude P. Muller, Sylvie Goossens, Fabrice Quet, Yves Buisson

**Affiliations:** 1 Institut de la Francophonie pour la Médecine Tropicale, Vientiane, Lao PDR; 2 Lao-Lux Laboratory, Institut Pasteur du Laos, Vientiane, Lao PDR; 3 Institute of Immunology, Centre de Recherche Public de la Santé / Laboratoire National de Santé, Dudelange, Luxembourg; 4 Independent consultant unaffiliated, Trégourez, France; CEA, FRANCE

## Abstract

**Background:**

During late 2012 and early 2013 several outbreaks of diphthe-ria were notified in the North of the Lao People’s Democratic Republic. The aim of this study was to determine whether the re-emergence of this vaccine-preventable disease was due to insufficient vaccination coverage or reduction of vaccine effectiveness within the affected regions.

**Methods:**

A serosurvey was conducted in the Huaphan Province on a cluster sampling of 132 children aged 12–59 months. Serum samples, socio-demographic data, nutri-tional status and vaccination history were collected when available. Anti-diphtheria and anti-tetanus IgG antibody levels were measured by ELISA.

**Results:**

Overall, 63.6% of participants had detectable diphtheria antibodies and 71.2% tetanus antibodies. Factors independently associated with non-vaccination against diphtheria were the distance from the health centre (OR: 6.35 [95% CI: 1.4–28.8], p = 0.01), the Lao Theung ethnicity (OR: 12.2 [95% CI:1,74–85, 4], p = 0.01) and the lack of advice on vac-cination given at birth (OR: 9.8 [95% CI: 1.5–63.8], (p = 0.01) while the level of maternal edu-cation was a protective factor (OR: 0.08 [95% CI: 0.008–0.81], p = 0.03). Most respondents claimed financial difficulties as the main reason for non-vaccination. Out of 55 children whose vaccination certificates stated that they were given all 3 doses of diphtheria-containing vaccine, 83.6% had diphtheria antibodies and 92.7% had tetanus antibodies. Furthermore, despite a high prevalence of stunted and underweight children (53% and 25.8%, respectively), the low levels of anti-diphtheria antibodies were not correlated to the nutritional status.

**Conclusions:**

Our data highlight a significant deficit in both the vaccination coverage and diphtheria vaccine effectiveness within the Huaphan Province. Technical defi-ciencies in the methods of storage and distribution of vaccines as well as unreliability of vac-cination cards are discussed. Several hypotheses are advanced to explain such a decline in immunity against diphtheria and recommendations are provided to prevent future outbreaks.

## Introduction

Lao People’s Democratic Republic (PDR) is a land-locked country in Southeast Asia. In 2011 the population was 6.2 million, with an under 5 mortality rate of 73 out of 1000 live births [1].

The Expanded Programme on Immunization (EPI) was introduced into the country in 1979 and aims to administer core vaccinations free of charge to all children under the age of 12 months, as recommended by the World Health Organization (WHO) (Table 1). It is mainly funded by several external donors including UNICEF, GAVI and others. Mobile outreach units have a remit to deliver vaccination services to villages four times per year. However, these services are facing challenges of funding, human resources and logistics. Therefore, EPI coverage in Lao PDR remains inadequate, mainly due to lack of resources and inaccessibility of remote populations. Data for the whole country from 2010–2011 show that approximately 78% of children receive all 3 doses of Diphtheria, Tetanus and Pertussis containing combination vaccine (DTP3) during the first year of life, with a large variability between different regions (100% in Vientiane Capital vs 60% in Borikhamxay) [1].

**Table 1:** Expanded Programme on Immunization schedule, Lao PDR.

Age	Vaccines
Birth	BCG, HepB0
6 weeks	DTP-Hib-HepB1, OPV1
10 weeks	DTP-Hib-HepB2, OPV2
14 weeks	DTP-Hib-HepB3, OPV3*
9–23 months	Measles/Rubella**

BCG: Bacillus Calmette-Guérin; DTP: Diphtheria, Tetanus, Pertussis; Hib: *Haemophilus influenzae* b; HepB: Hepatitis B; OPV: oral polio vaccine

* Pentavalent vaccine (HepB included since October 2009).

**Rubella included since 2012.

Due to the variable thermolability of vaccines, a break in the cold chain may lead to loss of potency [2]. In addition, some vaccinated individuals may have reduced response to vaccines, e.g. due to immune deficiencies, nutritional status and tolerance induced by maternal antibodies [3–6]. Importantly, malnutrition in Lao children is among the highest in the region [7,8]. Therefore, in addition to ensuring high vaccine coverage within populations and monitoring disease, it is important to assess the effectiveness of the vaccines following immunization.

Between October and mid-December 2012, the National Centre for Laboratory and Epidemiology (NCLE) reported 93 suspected cases of diphtheria, including 6 deaths, from the Xamtai and Huameuang districts, Huaphan province. Age distribution was specified for 24 suspected cases (29.2% under 4 years, 41.7% between 4 and 9 years, 20.8% between 10 and 14 years, 8.3% older than 14 years). Further outbreaks occurred in other provinces and continued in 2013 (about 29 suspected cases in Huaphan and 20 more nationwide in 2013). Such reemergence of a serious but vaccine- preventable disease could be due either to poor vaccination coverage or low effectiveness of the vaccines used.

The aim of this study was to evaluate the vaccine coverage and the post-vaccination immunity in children from two rural districts in Huaphan Province which reported most cases during the recent outbreaks.

## Methods

### Study type

**Fig 1:**
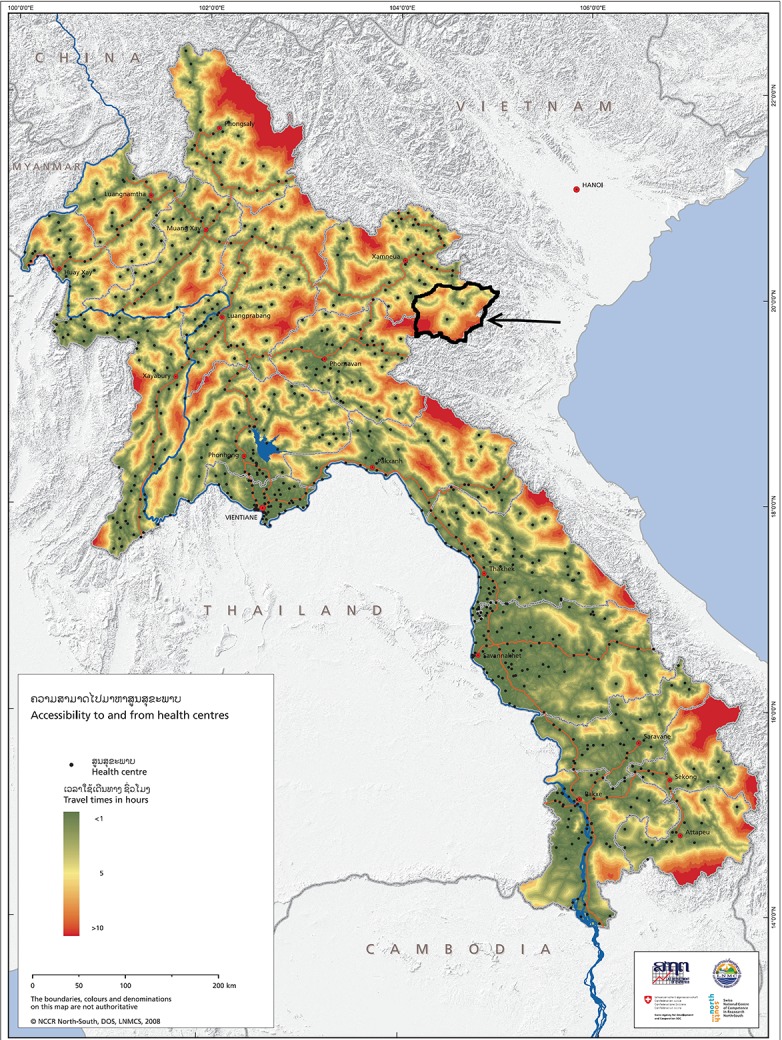
Study location site. The study area is outlined in black and indicated with an arrow. Map courtesy of the Swiss Agency for Cooperation and Development [11].

A cross-sectional study was conducted in two districts of Huaphan Province using a cluster sampling approach. The Huaphan Province is situated in the northeast of Lao PDR and borders Vietnam to the north, east and southeast (20.3333°N 103.833°E). The two districts targeted were Xamtai and Kuan, both located in the south of the province (Fig 1). District Xamtai was chosen because it is where the diphtheria outbreak began. District Kuan was chosen because it was a single district with Xamtai until June 2012 and therefore the children of the two districts had been vaccinated by the same vaccination team of Xamtai hospital. According to the Lao PDR census of 2005 [9], the total number of children aged 12–59 months in these two districts was 9758. In order to get a fairly accurate estimate, the theoretical sample size was calculated to be 370, for a confidence level of 95% and a confidence interval of 0.05, using the software OpenEpi [10]. The survey targeted 30 clusters of 13 people, i.e 390 people, leaving a margin in case of refusal to participate in the survey. Twenty-six villages (14 in the district of Xamtai and 12 in the district of Kuan) were randomly selected regardless of the district. Of these 26 villages, 13 were located within less than 100 minutes from the nearest health centre (12 in Xamtai and one in Kuan), using the most rapid means of transportation, i.e. by motorbike in the dry season [9]. The study was approved by the Laos National Ethics Committee (reference 2013–732).

### Study population

The objectives of the survey were explained to the villagers. Children aged 12 to 59 months whose parents or guardians were able to answer the questionnaire and give a written consent were recruited. The questionnaire included multiple choice questions distributed into four parts: demographic data, immunization status of the child, reasons for any non-vaccination and the use of health services [S1 Questionnaire]. When available, vaccination records in the form of “yellow cards” were used to determine the immunization status, in addition to parental recall. Acute malnutrition was determined by weight/height ratio and Middle Upper left Arm Circumference (MUAC) < -2 z-scores. Stunted and underweight participants were identified by height/age ratio < -2 z-scores and weight/age ratio < -2 z-scores, respectively [8].

### Serology

Sera were decanted on site and kept at 4°C for a maximum of 7 days, as recommended by the ELISA manufacturers until shipment by air to Vientiane capital, where they were stored at -80°C. Anti-diphtheria and anti-tetanus antibodies were measured with commercial ELISA kits (Verion/Serion, Germany and Euroimmun, Germany, respectively). According to the manufacturers' recommendations, anti-diphtheria antibody levels less than 0.1 international unit per milliliter (IU/ml) were considered negative or minimally protective, those between 0.1 to 1.0 IU/ml offered safe protection and those greater than 1.0 IU/ml gave long-term protection. For tetanus, antibody levels less than 0.1IU/ml were considered negative or uncertain, those between 0.1IU/ml and 0.5IU/ml indicated short-term protection, between 0.5IU/ml and 1.0IU/ml medium protection (booster required after 3 years) and above 1.0IU/ml long-term protection.

### Statistical analysis

Data collected were entered into a Microsoft Access database and analyzed using Stata Version 11.1 software. Qualitative variables were expressed as percentages with 95% confidence intervals (95% CI) and compared by Chi-square test or Fisher's exact test as appropriate. Quantitative variables were expressed as mean ± standard deviations (SD) and compared by t-tests or Mann Whitney according to the data characteristics. Factors associated with incomplete vaccination or no vaccination were analyzed by calculating the crude odds ratios (cOR) with 95% CI. Each independent variable with a p value ≤ 0.25 in bivariate analysis was included in multivariate analysis. Logistics step down was then performed from the initial regression model: all non-significant variables were removed from the original model in order of their significance. In the final model, only the factors independently associated with incomplete vaccination or no vaccination were retained. Results of bivariate and multivariate analyses were combined in the same table. For all statistical analyses the significance level was set at 5% (p <0.05).

## Results

### Socio-demographic characteristics of children

Of the 26 villages initially selected, only 22 could be visited due to logistical constraints, 10 located more and 12 located less than 100 minutes travel time from the next health centre. Furthermore, out of 415 parents informed about the study, only 132 gave consent for their child’s enrollment. The main reason given for non-participation was fear that the blood sampling would make their child ill. No further information was taken from those who did not wish to participate. Participants belonged to three main ethnic groups: Lao Loum/lowland Lao (59.8%), Lao Soung/ highland Lao (27.3%) and Lao Theung/slope-dweller (12.9%). Children were aged from 12 to 59 months (mean 38.7 ± 13.6 months) and 54.5% were male. The majority (62.1%) lived in villages located within 100 minutes from the nearest health facility. Most were born at home without medical assistance, especially in the remote villages >100 minutes from the health centres (98.0% vs. 78.0%; p = 0.002). Most fathers (89.3%) and mothers (97.7%) were farmers. There was no difference between remote and close villages in terms of parental educational level, ethnicity, or monthly income that did not exceed US $ 62 for 82% of households (Table 2). Respondents were either animist (73.5%) or buddhist (26.5%), with a higher percentage of animists in the less remote villages (84.2% vs. 56.0%, p<0.001).

**Table 2:** Socio-demographic characteristics of children.

Variables	Total (N = 132)	Village < 100 minutes (N = 82)	Village > 100 minutes (N = 50)	p
**Age (months)**				
Minimum	12.6	13.0	12.6	
Maximum	60.0	60.0	59.9	
Mean±SD	38.7±13.6	38.7±12.8	38.7±15.0	NS
**Gender N (%)**				
Male	72 (54.5)	44 (53.7)	28 (56.0)	NS
**Place of birth N (%)**				
Home	113.0 (85.6)	64.0 (78.0)	49.0 (98.0)	
District hospital	18.0 (13.6)	17.0 (20.8)	1.0 (2.0)	
Provincial hospital	1 (0.1)	1 (1.2)	0	0.002
**Ethnic group N (%)**				
Laoloum	79 (59.8)	43 (52.4)	36 (72)	
Laosoung	36 (27.3)	28 (34.2)	8 (16)	
Laotheung	17 (12.9)	11 (13.4)	6 (12)	NS
**Religion N (%)**				
Buddhist	35 (26.5)	13 (15.8)	22 (44.0)	
Animist	97 (73.5)	69 (84.2)	28 (56.0)	<0.001
**Father’s educational level N (%)**				
Illiterate	24 (18.3)	14 (17.1)	10 (20.5)	
Primary	76 (58)	47 (57.3)	29 (59.2)	
Secondary	25 (19.1)	17 (20.7)	8 (16.3)	NS*
**Mother’s educational level N (%)**				
Illiterate	69 (52.3)	42 (51.2)	27 (54)	
Primary	50 (37.9)	29 (35.4)	21 (42)	
Secondary	13 (9.8)	11 (13.4)	2 (4)	NS
**Father’s occupation N (%)**				
Civil servant	11 (08.4)	09 (11)	2 (4.1)	
Farmer	117 (89.3)	71 (86.6)	46 (93.9)	
Worker	3 (02.3)	02 (2.4)	1 (2)	NS
**Mother’s occupation N (%)**				
Tradeswoman	1 (0.76)	0	1 (2)	
Farmer	129 (97.7)	81 (98.8)	48 (96)	
Worker	1 (0.76)	0	1 (2)	NS
Monthly income (LAK)** N (%)				
< 100 000	16 (12.1)	7 (8.5)	9 (18)	
100 000–300 000	74 (56.1)	48 (58.6)	26 (52)	
300 000–500 000	24 (18.2)	16 (19.5)	8 (16)	
> 500 000	18 (13.6)	11 (13.4)	7 (14)	NS

*NS = not significant (p ≥ 0.05)

**Lao kips (8000 LAK worth one US dollar).

### Immunization status of children

**Fig 2:**
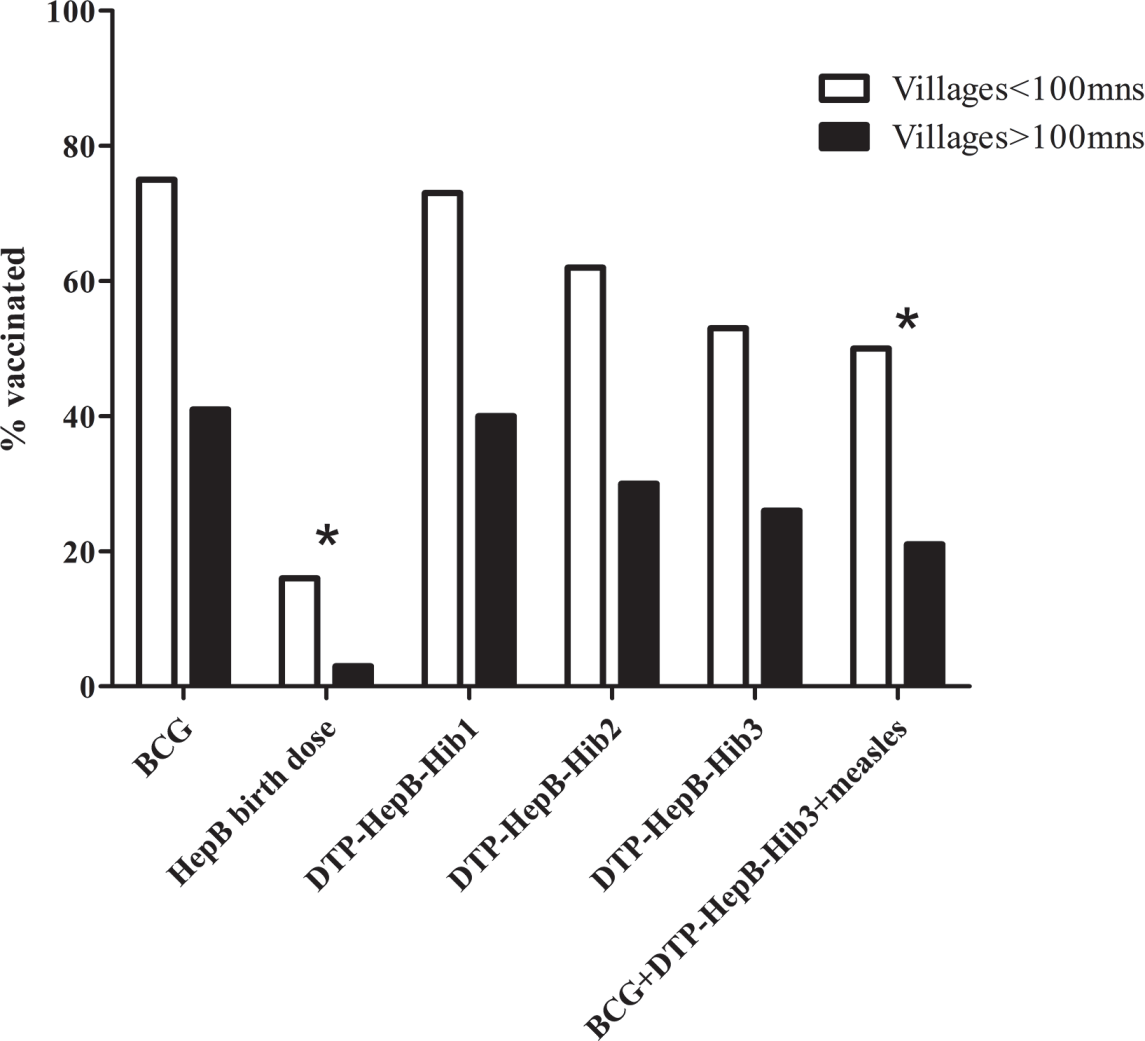
Near and remote village vaccination history according to yellow cards and interview (n = 132; mns = minutes; *p<0.05).

Based on vaccination certificates, so called “yellow cards” (n = 66) or, if not available, on parents’ responses (n = 66), the rates of immunization coverage with DTP-containing vaccine were 85.6% for the first dose, 69.7% for the second dose, and only 59.8% for the third dose. Children of villages close to health centres were more often fully vaccinated against BCG, DTP, polio and measles than those of remote villages (61% vs. 42%, p = 0.03; Fig 2).

The vaccination coverage rates for each of the three DTP injections were significantly higher in children whose vaccination status was known from the yellow card than among children without card (p <0.0001, Table 3).

**Table 3:** Vaccination status of children according to the source of information.

Vaccinations performed	Total N = 132 (%)	Vaccination card N = 66 (%)	Parental recall N = 66 (%)	p
BCG	116 (87.9)	65 (98.5)	51 (77.3)	0.2
HepB at birth	19 (14.4)	14 (21.2)	5 (7.6)	0.2
(DTP HepB +Hib +Polio) 1	113 (85.6)	66 (100)	47 (71.2)	<0.0001
(DTP HepB +Hib +Polio) 1+2	92 (69.7)	57 (86.3)	35 (53.0)	<0.0001
(DTP HepB+Hib+Polio) 1+2+3	79 (59.8)	55 (83.3)	24 (36.4)	<0.0001

BCG: Bacillus Calmette-Guérin; HepB: Hepatitis B; DTP: Diphtheria, Tetanus, Pertussis; Hib: *Haemophilus influenzae* b.

### Serological status of children

**Fig 3:**
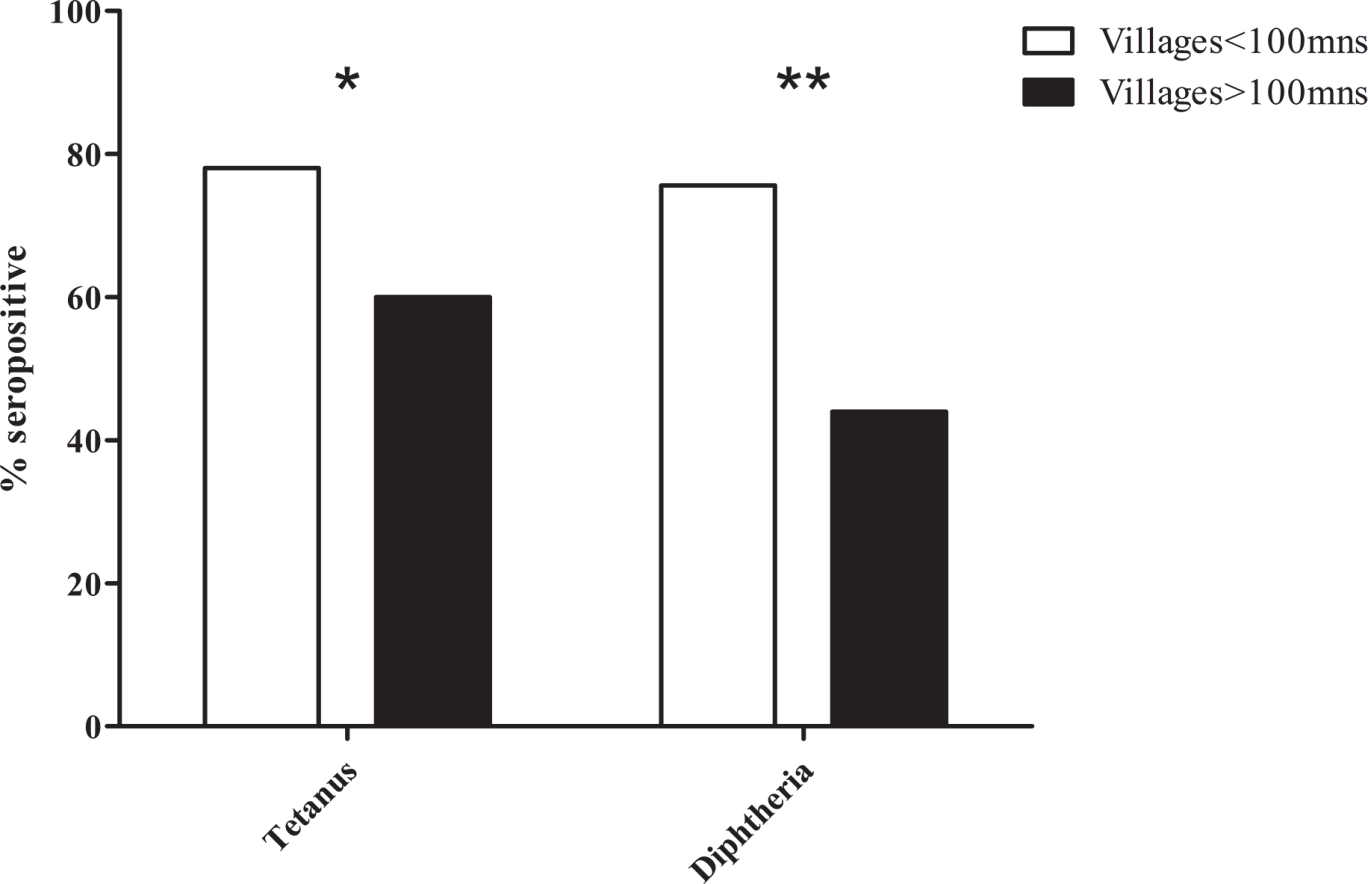
Anti-diphtheria and anti-tetanus antibody seroprevalence in children from villages less than or greater than 100 minutes travel from health centres (n = 132; mns = minutes; *p = 0.02, **p<0.001).

Diphtheria antibodies were detected in 84 out of the 132 children (63.6%) including 58 (43.9%) with a titer greater than 1 IU/ml, corresponding to long-term protection. The prevalence of these protective antibodies was higher among children in villages close to health centers than for children in remote villages (75.6% versus 44%, p<0.001; Fig 3).

Tetanus antibodies were detected in 94 children (71.2%) including 76 (57.6%) with long-term protection. Again, the prevalence of these antibodies was higher in the villages close to health centers than in remote villages (78.1% vs. 60%; p = 0.02; Fig 3).

Out of the 55 children who received the 3 doses of DTP-Hib vaccine as evidenced by their yellow card, 46 (83.6%) and 51 (92.7%) reacted positively for diphtheria and tetanus antibodies, respectively (Table 4).

**Table 4:** Seroprevalence of diphtheria and tetanus antibodies according to the number of DTP vaccine doses (data from children who provided vaccination records).

Vaccines listed	Children with yellow cards N = 66 (%)	Children with anti-diphtheria antibodies N (%)	Children with anti-tetanus antibodies N (%)
DTP-HepB (1)	9 (13.6)	3 (33.3)	3 (33.3)
DTP-HepB (1+2)	2 (3.0)	2 (100)	2 (100)
DTP- HepB (1+2+3)	55 (83.3)	46 (83.6)	51 (92.7)

DTP: Diphtheria, Tetanus, Pertussis; HepB: Hepatitis B.

### Factors affecting incomplete vaccination or non-vaccination of children

All the 61 parents in charge of partially vaccinated or unvaccinated children agreed to answer questions about the factors impacting adherence to EPI. In multivariate analysis, ethnicity was the only factor independently associated with partial immunization (Table 5): Lao Loum children were better vaccinated than Lao Soung (p = 0.03) and Lao Theung children (p = 0.04). For the 15 children (11.4%) who received no vaccine (Table 6), three factors were independently associated with the absence of vaccination: the distance from home to the health centre (OR: 6.35 [95% CI: 1.4–28.8], p = 0.01), the Lao Theung ethnicity (OR: 12.2 [95% CI: 1,74–85, 4], p = 0.01) and the lack of advice on vaccination given at birth (OR: 9.8 [95% CI: 1.5–63.8], p = 0.01) while the level of maternal education was the only protective factor (OR: 0.08 [95% CI: 0.008–0.81], p = 0.03).

**Table 5:** Logistical regression; factors associated with incomplete vaccination of children.

Independent variables	Bivariate analysis			Multivariate analysis		
**Father's occupation**	cOR	95% CI	p	aOR	95% CI	p
Civil servant (ref)	-	-	-	-	-	-
Farmer	2.61	[0.54–12.66]	0.23	0.55	[0.025–11.9]	0.7
**Father's educational level**						
Illiterate (ref)	-	-	-	-	-	-
Primary	0.46	[0.18–1.17]	0.1	0.35	[0.11–1.05]	0.06
Secondary	0.47	[0.15–1.5]	0.2	0.62	[0.14–2.75]	0.5
Technical college	0.2	[0.02–1.98]	0.17	0.15	[0.003–7.53]	0.3
**Mother's educational level**						
Illiterate (ref)	-	-	-	-	-	-
Primary	1.08	[0.51–2.29]	0.8	2.1	[0.83–5.37]	0.1
Secondary	0.32	[0.06–1.56]	0.16	1.8	[0.19–16.19]	0.6
**Ethnic group**						
Laoloum (ref)	-	-	-	-	-	-
Laosoung	2.36	[1.03–5.41]	0.04	2.9	[1.06–7.85]	0.03
Laotheung	4.21	[1.42–12.55]	0.01	3.4	[1.02–11.29]	0.04
**Religion**						
Buddhist (ref)	-	-	-	-	-	-
Animist	0.87	[0.39–1.95]	0.7	-	-	-
**Time to reach the nearest health center (minutes)**						
<100 (ref)	-	-	-	-	-	-
>100	1.65	[0.79–3.43]	0.18	1.6	[0.67–3.77]	0.3
**Sex of child**						
Male (ref)	-	-	-	-	-	-
Female	1.15	[0.56–2.37]	0.7	-	-	-
**Advice on immunization received at birth**						
Yes (ref)	-	-	-	-	-	-
No	1.54	[0.73–3.23]	0.26	-	-	-

ref: reference; cOR: crude Odds ratio; aOR: adjusted Odds ratio; 95% CI: 95% confidence interval.

**Table 6:** Logistical regression; factors associated with non-vaccination of children.

Independent variables	Bivariate analysis			Multivariate analysis		
	cOR	95% CI	p	aOR	95% CI	p
**Father’s educational level**						
Illiterate (ref)	-	-	-	-	-	-
Primary	0.85	[0.24–2.95]	0.7	-	-	-
**Mother's educational level**						
Illiterate (ref)	-	-	-	-	-	-
Primary	0.08	[0.01–0.63]	0.01	0.08	[0.008–0.81]	0.03
**Ethnic group**						
Laoloum (ref)	-	-	-	-	-	-
Laosoung	11	[2.2–54.9]	0.003	1.73	[0.23–13.18]	0.5
Laotheung	16	[2.8–92.2]	0.002	12.2	[1.74–85.4]	0.01
**Religion**						
Buddhist (ref)	-	-	-	-	-	-
Animist	5.7	[0.72–45.3]	0.09	7.59	[0.36–159.7]	0.2
**Time to reach the nearest health center (minutes)**						
<100 (ref)	-	-	-	-	-	-
>100	2.04	[0.69–6.02]	0.1	6.35	[1.4–28.8]	0.01
**Sex of child**						
Male (ref)	-	-	-	-	-	-
Female	0.78	[0.26–2.32]	0.6	-	-	-
**Advice on immunization received at birth**						
Yes (ref)	-	-	-	-	-	-
No	9.7	[2.6–36.8]	0.001	9.8	[1.5–63.8]	0.01

ref: reference; cOR: crude Odds ratio; aOR: adjusted Odds ratio; 95% CI: 95% confidence interval.

Lack of money was the main reason for non-vaccination advanced by responders (63.9%). Difficulties to get their children vaccinated in the absence of mobile team were claimed by 78.1% of people in villages near health centers vs 48.3% of people in remote villages. Other reasons given for non-vaccination were ignorance of where children could be immunized, the perception of worthlessness and the fear of vaccine side effects.

### Correlations between post-vaccine immunity and nutritional status

The prevalence of acute malnutrition estimated by weight/height ratio and upper left arm circumference was 3% and 9.1%, respectively. Fifty three percent of the children were stunted and 25.8% underweight without statistical difference between nearby and remote villages. No significant association was found between post-vaccination antibody levels and nutritional indices, except a higher rate of tetanus antitoxins in underweight children (p = 0.01, Table 7).

**Table 7:** Seroprevalence of diphtheria and tetanus antibodies according the nutritional status of children.

Nutritional status	Diphtheria antibodies			Tetanus antibodies		
	yes = 84	no = 48	p	yes = 94	no = 38	p
**Malnutrition** (W/H ratio < -2 z-scores)	4 (48)	0	NS	4 (4.3)	0	NS
**Malnutrition** (MUAC < -2 z-scores)	8 (9.5)	4 (8.3)	NS	9 (9.6)	3 (7.9)	NS
**Stunting**	48 (57.1)	22 (45.8)	NS	54 (57.5)	16 (42.1)	NS
**Underweight**	25 (29.8)	9 (18.8)	NS	30 (31.9)	4 (10.5)	0.01

W/H ratio: weight-for-height ratio; MUAC: Middle Upper Arm Circumference.

## Discussion

The occurrence of a diphtheria epidemic in the 21st century is an unacceptable event that should draw the attention of public health services. Hence the main objective of this survey was to assess the vaccine coverage and the prevalence of post-vaccinal diphtheria antibodies in the child population of the Huaphan Province, Lao PDR, hit by a diphtheria outbreak in December 2012.

We observed that the overall rate of immunization coverage for three doses of DTP was only 59.8%, which is well below the national target of 90% [11,12]. However, there was a major difference between the rates observed in children whose yellow card could be shown (83.3%) and children without yellow card (36.4%). This very low rate, only obtained from questionnaires, may reflect a bias of understanding or recall resulting in underestimation of the true vaccination coverage. A similar difference was found in an Indian study with a vaccination coverage rate significantly higher after analyzing vaccination cards than on the basis of parents recall. However, their study found an overestimation of the coverage calculated from immunization cards due to multiple sources of registration, duplication of entries, lack of crosschecks, possible errors in data collection and management and exaggerated coverage reports by local health authorities [14].

A major determinant is the difficulty for mobile vaccination units to reach the target populations. Huaphan province is part of the mountainous regions where many villages are not accessible by road during the rainy season. Thus, a recent supervision of EPI in Lao PDR recommended increasing the number of rounds to at least six per year in order to increase the vaccination coverage [13]. A nationwide study [13] and a study in Oudomxay [15] on the causes of non-vaccination have both demonstrated a significant relationship with distance from the nearest health care centre. Our study confirms the importance of the remoteness of villages over 100 minutes from the health center as a key factor for non-vaccination. In addition, as more than 90% of parents are farmers, their children often accompany them in the fields, hence their absence during immunization campaigns. The main cause for non-immunization or incomplete immunization of children was their difficulty to access to the healthcare centres. This is why lack of money is often claimed by responders as a reason for non-vaccination. Although vaccination is done for free, the cost of travel and the loss of a day's work represent a financial handicap for these poor people. Other reasons most often cited were the perceived uselessness of vaccines, the ignorance of where to be vaccinated and the fear of side effects. These data corroborate the results of two recent studies regarding reasons for non-vaccination in Lao PDR [12,16]. We must also consider the absence of advice given at birth as a factor independently associated with non-vaccination of children, from the fact that 85.6% of births took place at home. Multivariate analysis identified two additional factors inversely correlated with the children’s immunization: the low level of maternal education and belonging to an ethnic minority (Lao Soung and Lao Theung). The impact of education level of mothers on the vaccination coverage of children has already been highlighted in many studies [17–20]. However, belonging to an ethnic minority is less often cited. As in many developing countries, these groups are often marginalized and do not have equitable access to health services [17–21].

How to explain such a low prevalence of post-vaccination antibodies? Protective concentrations of diphtheria antitoxins were detected in only 63.6% of children tested. The most likely reason is the poor DTP vaccination coverage found in this population. However, out of the 55 children who received three doses of DTP vaccine as evidenced by the yellow card, 83.6% had protective antibodies against diphtheria and 92.7% against tetanus. Three hypotheses can be advanced to explain such discrepancy: a poor quality of injected vaccines, a poor immune response of children, or a lack of reliability of the yellow card.

Although unlikely, the possibility of bad batches of vaccine imported by the Ministry of Health cannot be discounted and requires further investigation. In addition, several steps of the supply chain in vaccination campaigns may compromise vaccine quality (conditions of storage, cold chain breaks during transport, etc.) [22]. However, diphtheria and tetanus toxoids are among the most stable vaccines and can be stored for several months at room temperature and for several weeks at 37°C [23]. The currently used vaccine in Lao PDR is diphtheria–tetanus–whole cell pertussis–hepatitis B–*Haemophilus influenzae* type b (DTPw–HepB–Hib) combination vaccine, supplied by Crucell/Berna Biotech. Although this vaccine is deemed immunogenic when given in a timely manner [24], a recent study highlighted a low immunogenicity against hepatitis B in Senegalese children, with a low anti-diphtheria response significantly associated with the lack of anti-HBs antibodies [25]. However, this study did not rule out the possible role of malnutrition. Indeed, the possibility of an impaired immune response to the vaccine should also be considered. It may be related to nutritional status, to the presence of concurrent infections (viruses, intestinal parasites, etc.), or to immune tolerance due to the persistence of maternal antibodies [6, 26–28]. The prevalence of stunting in Asia is high, related to poor nutritional status and health care of mothers during pregnancy, resulting in intrauterine growth retardation and a low birth weight [29]. Notably, Lao PDR has one of the highest rates of malnutrition in Southeast Asia [7]. Yet, despite a 53% rate of stunting in the study population, we could not highlight a relationship between nutritional status and lack of humoral response to vaccination. Finally, as mentioned above, the reliability of vaccination cards must also be questioned and should be evaluated by an independent survey [13, 30, 31].

Notably, the prevalence of antibodies against tetanus was higher than against diphtheria. This may reflect an unequal immunogenicity between the two toxoids. It is also possible that the ELISA used to measure diphtheria antibodies had a sensitivity lower than that used for tetanus antibodies, as a recent study revealed a large variation between the tests according to the manufacturer [32]. Further studies are warranted to investigate this observation.

Given the fragility of immunization coverage data obtained from questionnaires from parents’ recall, even in possession of a vaccination card, the main strength of this study is the determination of protective antibodies elicited by vaccination. This is probably the most reliable method to assess the true protection conferred by diphtheria and tetanus toxoids. Serological surveys can provide a valuable complement to immunization coverage surveys in low- and middle-income countries [13, 30]. However, this study is limited by the small sample size and the fact that many parents refused to participate. This attitude of distrust and fear with respect to health officials is still common among people from ethnic minorities [33]. This can result in a selection bias, but we can infer that the immunization coverage rates are likely lower among families that have not given their consent.

The occurrence of a diphtheria epidemic in the Huaphan Province clearly reveals a serious deficiency in immunization coverage of children under 5 years of age. Whether significant proportions of children targeted by the EPI are not reached by mobile teams or the vaccines are poorly preserved and used, or that the immunogenicity may have been low, remains to be determined more precisely but the first two factors appear to be associated predominantly.

## Conclusion

This study supports the findings of previous surveys conducted in Lao PDR leading to the following recommendations: strengthening health education for target populations, prioritization of vaccination in the national health strategies, reinforcement of mobile teams in remote areas, improving coordination with village leaders to reach all target children, strengthening of equipment of all health centers (fridges, kerosene and/or gas), ensuring regular supply of vaccines, cold chain management and adoption of temperature-monitoring technologies during storage and transport of vaccines. With regard to ethnic minorities, an anthropological approach should be implemented to take into account any cultural barriers to vaccination. The implementation of these recommendations should be a priority in the most remote provinces in order to avoid the recurrence of outbreaks of deadly but vaccine preventable diseases in children.

## Supporting Information

S1 Questionnaire (DOC)

## References

[1] Department of Planning and International Cooperation M, Lao PDR. National Health Statistic Report FY 2010–2011: contributing to monitor Millenium Development Goals. 2011.

[2] Kristensen D, Chen D, Cummings R. Vaccine stabilization: research, commercialization, and potential impact. Vaccine. 2011;29: 7122–7124. 10.1016/j.vaccine.2011.05.070 21651941

[3] Lalor MK, Floyd S, Gorak-Stolinska P, Weir RE, Blitz R, Branson K et al BCG vaccination: a role for vitamin D? PLoS One. 2011; 6: e16709 10.1371/journal.pone.0016709 21304967 PMC3031626

[4] Okwo-Bele JM, Cherian T. The expanded programme on immunization: a lasting legacy of smallpox eradication. Vaccine. 2011;29 (Suppl 4): D74–79. 10.1016/j.vaccine.2012.01.080 22486980

[5] Kaufman DR, De Calisto J, Simmons NL, Cruz AN, Villablanca EJ, Mora JR et al Vitamin A deficiency impairs vaccine-elicited gastrointestinal immunity. J Immunol. 2011;187: 1877–1883. 10.4049/jimmunol.1101248 21765014 PMC3150351

[6] Siegrist CA. Mechanisms by which maternal antibodies influence infant vaccine responses: review of hypotheses and definition of main determinants. Vaccine. 2003;21: 3406–3412. 12850349 10.1016/s0264-410x(03)00342-6

[7] Kamiya Y. Socioeconomic determinants of nutritional status of children in Lao PDR: effects of household and community factors. J Health Popul Nutr. 2011;29: 339–348. 21957672 10.3329/jhpn.v29i4.8449 PMC3190364

[8] Barennes H, Simmala C, Odermatt P, Thaybouavone T, Vallee J, Martinez-Aussel B et al Postpartum traditions and nutrition practices among urban Lao women and their infants in Vientiane, Lao PDR. Eur J Clin Nutr. 2009;63: 323–331. 18000519 10.1038/sj.ejcn.1602928 PMC3435433

[9] National Statistics Centre of the Lao PDR. Population Census (2005) Available: http://www.nsc.gov.la/Products/Populationcensus2005/PopulationCensus2005_chapter1.htm

[10] Sullivan KM, Dean A, Soe MM (2009) OpenEpi: a web-based epidemiologic and statistical calculator for public health. Public Health Rep 124:471–4. 19445426 10.1177/003335490912400320 PMC2663701

[11] Messerli P, Heinimann A, Epprecht M, Phonesaly S, Thiraka C, Minot N, editors. 2008: Socio-Economic Atlas of the Lao PDR—an Analysis based on the 2005 Population and Housing Census Swiss National Center of Competence in Research (NCCR) North-South, University of Bern, Bern and Vientiane: Geographica Bernensia.

[12] Kitamura T, Komada K, Xeuatvongsa A, Hachiya M . Factors affecting childhood immunization in Lao People's Democratic Republic: a cross-sectional study from nationwide, population-based, multistage cluster sampling. Biosci Trends. 2013;7: 178–185. 24056168

[13] WHO. International review of the Expanded Programme on Immunization in the Lao People’s Democratic Republic, May 2012. http://www.wpro.who.int/immunization/documents/intl_revw_epi_lao/en/ (accessed January 4, 2015).

[14] Babu G, Olsen J, Jana S, Nandy S, Farid M, Sadhana. Evaluation Of Immunization Cards And Parental Recall Against Gold Standard For Evaluating Immunization Coverage. Internet Journal of Epidemiology. 2011;9.

[15] Maekawa M, Douangmala S, Sakisaka K, Takahashi K, Phathammavong O, Xeuatvongsa A et al Factors affecting routine immunization coverage among children aged 12–59 months in Lao PDR after regional polio eradication in western Pacific region. Biosci Trends. 2007;1: 43–51. 20103866

[16] Phimmasane M, Douangmala S, Koffi P, Reinharz D, Buisson Y. Factors affecting compliance with measles vaccination in Lao PDR. Vaccine. 2010;28: 6723–6729. 10.1016/j.vaccine.2010.07.077 20692220

[17] Streatfield K, Singarimbun M, Diamond I. Maternal education and child immunization. Demography. 1990;27: 447–455. 2397822

[18] Bhuiya A, Bhuiya I, Chowdhury M. Factors affecting acceptance of immunization among children in rural Bangladesh. Health Policy Plan. 1995;10: 304–312. 10151848 10.1093/heapol/10.3.304

[19] Xie J, Dow WH. Longitudinal study of child immunization determinants in China. Soc Sci Med. 2005;61: 601–611. 15899319 10.1016/j.socscimed.2004.12.016

[20] Parashar S. Moving beyond the mother-child dyad: women's education, child immunization, and the importance of context in rural India. Soc Sci Med. 2005;61: 989–1000. 15955401 10.1016/j.socscimed.2004.12.023

[21] Chowdhury AM, Bhuiya A, Mahmud S, Abdus Salam AK, Karim F. Immunization divide: who do get vaccinated in Bangladesh? J Health Popul Nutr. 2003;21: 193–204. 14717565

[22] Rogers B, Dennison K, Adepoju N, Dowd S, Uedoi K. Vaccine Cold Chain: Part 2. Training personnel and program management. AAOHN J. 2010;58: 391–400. 10.3928/08910162-20100816-02 20795579

[23] Galazka A, Milstein J, Zaffran M. Thermostability of vaccines. WHO document WHO/GPV/9807 Geneva: World Health Organization, 1998.

[24] Kanra G, Kara A, Demiralp O, Contorni M, Hilbert AK, Spyr C et al Safety and immunogenicity of a new fully liquid DTPw-HepB-Hib combination vaccine in infants. Hum Vaccin. 2006;2: 155–160. 17012890 10.4161/hv.2.4.2942

[25] Rey-Cuille MA, Seck A, Njouom R, Chartier L, Sow HD, Mamadou et al Low immune response to hepatitis B vaccine among children in Dakar, Senegal. PLoS One. 2012;7: e38153 10.1371/journal.pone.0038153 22666468 PMC3364238

[26] Chan J, Nirwati H, Triasih R, Bogdanovic-Sakran N, Soenarto Y, Hakimi M et al Maternal antibodies to rotavirus: could they interfere with live rotavirus vaccines in developing countries? Vaccine. 2011;29: 1242–1247. 10.1016/j.vaccine.2010.11.087 21147127

[27] Gans HA, Maldonado YA. Loss of passively acquired maternal antibodies in highly vaccinated populations: an emerging need to define the ontogeny of infant immune responses. J Infect Dis. 2013;208: 1–3. 10.1093/infdis/jit144 23661801

[28] Labeaud AD, Malhotra I, King MJ, King CL, King CH. Do antenatal parasite infections devalue childhood vaccination? PLoS Negl Trop Dis. 2009;3: e442 10.1371/journal.pntd.0000442 19478847 PMC2682196

[29] Khor GL. Update on the prevalence of malnutrition among children in Asia. Nepal Med Coll J. 2003;5: 113–122. 15024783

[30] Cutts FT, Izurieta HS, Rhoda DA. Measuring coverage in MNCH: design, implementation, and interpretation challenges associated with tracking vaccination coverage using household surveys. PLoS Med. 2013;10: e1001404 10.1371/journal.pmed.1001404 23667334 PMC3646208

[31] Sychareun V, Hansana V, Phengsavanh A, Chaleunvong K, Eunyoung K, Durham J. Data verification at health centers and district health offices in Xiengkhouang and Houaphanh Provinces, Lao PDR. BMC Health Serv Res. 2014;14: 255 10.1186/1472-6963-14-255 24929940 PMC4118319

[32] Zasada AA, Rastawicki W, Smietanska K, Rokosz N, Jagielski M. Comparison of seven commercial enzyme-linked immunosorbent assays for the detection of anti-diphtheria toxin antibodies. Eur J Clin Microbiol Infect Dis. 2013;32:891–7. 10.1007/s10096-013-1823-y 23354678

[33] Scheppers E, van Dongen E, Dekker J, Geertzen J, Dekker J. Potential barriers to the use of health services among ethnic minorities: a review. Fam Pract. 2006;23:325–48. 16476700 10.1093/fampra/cmi113

