# Supplementary material for: Diphtheria in Lao PDR: Insufficient Coverage or Ineffective Vaccine?
Source: PLoS One. 2015 Apr 24;10(4):e0121749. doi: 10.1371/journal.pone.0121749 (PMC4409043; doi:10.1371/journal.pone.0121749)
Supplement: S1 Questionnaire — (DOC) [file pone.0121749.s001.doc]

**QUESTIONNAIRE**

**(for parents or caretakers)**

Questionnaire N° /________/ :

Date :……………………

Village : …………………...District: *1. Xamtai 2. Kuan*

**Person questioned:** *1=father    2=mother   3=grandfather    4=grandmother*

*5=person in charge 6=other……………*

1. **GENERAL INFORMATION**
2. **About children**

**Q01. Last name:** ….................................................................................

**Q02. First name**:……………………………………………………

**Q03. Date of birth**: ………/………/………

**Q04. Gender**                                   *1 = male 2 = female*

**Q05. Weight**……………………………........…………….… kg

**Q06. Height** ……………………………............................... cm

**Q07. Middle Upper Arm Circumference**: …...............…… cm

**Q08. Presence of edema**

*0= no                       1= yes*

**Q9. Breast-feeding**

*0= no                       1= yes*

***If so until what age?***.................................. (Months)

**Q10. Place of birth**

*1=home       2=* *health center 3=district hospital 4=provincial hospital    6=other……………..*

1. **About parents**

**Q11. Father’s occupation:**

*1=unemployed    2=civil servant    3=* *tradesman   4=farmer 5=worker*

*6=other..........................*

**Q12. Father's date of birth:** …......./.........../...............

**Q13. Father’s educational level:**

*1=illiterate     2=primary   3=secondary   4=technical college 5=university*

*6=other .....................................................*

**Q14. Mother’s occupation:**

*1=unemployed    2=civil servant    3=* *tradeswoman   4=farmer 5=worker*

*6=other..........................*

**Q15. Mother's date of birth:** …......./.........../...............

**Q16. Mother’s educational level:**

*1=illiterate     2=primary   3=secondary   4=technical college 5=university*

*6=other .....................................................*

**Q17. Ethnic group:**

*1=laoloum    2=laosoung      3=laotheung*

**Q18. Religion**

*1=buddhist     2=christian      3=animist    4=other* …………………

**Q19. Number of persons in household:** ………………

**Q20. Number of children under 5 years:** ………………

**Q21. Number of people working:** …...............................

**Q22. Monthly income** …................................................

**II. VACCINATION STATUS OF CHILDREN**

**Q23. Have you received any advice on vaccination at the child's birth?**

*0=no                       1=yes*

**Q24. Did you vaccinate your child? (if not, fill part III)**

*0=no                       1=yes*

**Q25. Where was he/she vaccinated?**

*1=village 2=health center 3=district hospital*

*4=provincial hospital 5=central hospital     6=other…………….*

**Q26. Vaccination record available (yellow card)** 

*0=no                       1=yes*

**Q27. Vaccines recommended by EPI (according to the yellow card)**

|  | **vaccines** | **Yes/ No** | **Date** |
| --- | --- | --- | --- |
| at birth | BCG |  |  |
|  | HepB |  |  |
| 6 weeks | DTC-HepB-Hib1 |  |  |
|  | Polio1 |  |  |
| 10 weeks | DTC-HepB-Hib2 |  |  |
|  | Polio2 |  |  |
| 14 weeks | DTC-HepB-Hib3 |  |  |
|  | Polio3 |  |  |
| 9 months | Measles |  |  |

**Q28. Fully immunized according to the age? (if yes, fill part IV)**

*0=no                        1=yes*

**III. REASONS FOR NON-VACCINATION**

**(for people whose child is not fully immunized)**

**Q29. Don't know where to go to get vaccination (lack of information)**

*0=no                        1=yes*

**Q30. Think that vaccines are not free**

*0=no                        1=yes*

**Q31. Have difficulties to move**

*0=no                        1=yes*

**Q32. Lack of time**

*0=no                        1=yes*

**Q33. Think vaccines are useless**

*0=no                        1=yes*

**Q34. Fear of the needle**

*0=no                        1=yes*

**Q35. Think vaccinations are done at school**

*0=no                        1=yes*

**Q36. Fear of side effects**

*1=paresis       2=fever         3=convulsions     4=other…………………….*

**Q37. Negative rumors about vaccination**

*0=no                      1=yes*

**Q38. Other reasons**

specify ……………………………………………………

**IV. SOURCES OF INFORMATION ABOUT VACCINATION AND WELCOME TO THE VACCINATION CENTER**

**Q39. Do you know about vaccinations?**   (if no, go to question 41)

*0=no                       1=yes*

**Q40. From whom did you get information regarding vaccination?**

*1=doctor     2=mobile team    3= family     4*=*professional environment      5=media*

*6=other …………………………….*

**Q41. How long did you wait to have your child vaccinated?**

*0= < 30 minutes        1= 30 min-1 hour        2= >1-2 hours         3= > 2 hours*

**Q42. Are the opening hours of the vaccination center appropriate?**

*0=no 1=yes*
